# Prevention of Covid-19 Infection and Related Complications by Ozonized Oils

**DOI:** 10.3390/jpm11030226

**Published:** 2021-03-22

**Authors:** Alberto Izzotti, Enzo Fracchia, William Au, Monica Colombo, Ulrich Pfeffer, Laura Emionite, Simone Pavan, Daniele Miotto, Paola Lova, Elena Grasselli, Emanuela Faelli, Ruggeri Piero, Micaela Tiso, Alessandra Pulliero

**Affiliations:** 1 Department of Experimental Medicine, University of Genoa, 16132 Genoa, Italy; emanuela.faelli@unige.it (E.F.); ruggeri@unige.it (R.P.); 2 IRCCS Ospedale Policlinico San Martino, 16132 Genoa, Italy; monica.colombo@hsanmartino.it (M.C.); ulrich.pfeffer@hsanmartino.it (U.P.); laura.emionite@hsanmartino.it (L.E.); 3 Galliera Hospital, 16132 Genoa, Italy; enzo.fracchia@galliera.it; 4 Faculty of Medicine, Pharmacy, Science and Technology, The George Emil Palade University, 540142 Targu Mures, Romania; wau@stu.edu.cn; 5 BWH Graphic Solutions, 28001 Madrid, Spain; pavan@bwhenergysolutions.com (S.P.); miotto@bwhenergysolutions.com (D.M.); 6 Department of Chemistry and Industrial Chemistry, University of Genoa, 16132 Genoa, Italy; paola.lova@unige.it; 7 Department of Earth Sciences, University of Genoa, 16132 Genoa, Italy; elena.grasselli@unige.it; 8 MICAMO Spin-Off Department of Earth Sciences, University of Genoa, 16132 Genoa, Italy; micaela.tiso@unige.it; 9 Department of Health Sciences, University of Genoa, 16132 Genoa, Italy; alessandra.pulliero@unige.it

**Keywords:** COVID-19, SARS-CoV-2, chemoprophylaxis, prevention, oxidative stress, COVID-19 challenge test

## Abstract

Background: The COVID-19 pandemic continues to ravage the human population; therefore, multiple prevention and intervention protocols are being rapidly developed. The aim of our study was to develop a new chemo-prophylactic/-therapeutic strategy that effectively prevents COVID-19 and related complications. Methods: In in vitro studies, COVID-19 infection-sensitive cells were incubated with human oropharyngeal fluids containing high SARS-CoV-2 loads. Levels of infection were determined via intra-cellular virus loads using quantitative PCR (qPCR). Efficacies for infection prevention were determined using several antiviral treatments: lipid-encapsulated ozonized oil (HOO), water-soluble HOO (HOOws), UV, and hydrogen peroxide. In in vivo studies, safety and efficacy of HOO in fighting COVID-19 infection was evaluated in human subjects. Results: HOO in combination with HOOws was the only treatment able to fully neutralize SARS-CoV-2 as well as its capacity to penetrate and reproduce inside sensitive cells. Accordingly, the feasibility of using HOO/HOOws was tested in vivo. Analysis of expired gas in healthy subjects indicates that HOO administration increases oxygen availability in the lung. For our human studies, HOO/HOOws was administered to 52 cancer patients and 21 healthy subjects at high risk for COVID-19 infection, and all of them showed clinical safety. None of them developed COVID-19 infection, although an incidence of at least 11 cases was expected. Efficacy of HOO/HOOws was tested in four COVID-19 patients obtaining recovery and qPCR negativization in less than 10 days. Conclusions: Based on our experience, the HOO/HOOws treatment can be administered at standard doses (three pills per day) for chemo-prophylactic purposes to healthy subjects for COVID-19 prevention and at high doses (up to eight pills per day) for therapeutic purposes to infected patients. This combined prevention strategy can provide a novel protocol to fight the COVID-19 pandemic.

## 1. Introduction

With waves and waves of COVID-19 infections around the world, it is urgent to develop novel and effective prevention and intervention programs against the pandemic and as rapidly as possible. The aim of our study was, therefore, to develop a novel chemo-prophylactic approach which would generate immediate preventive efficacy and would also have therapeutic capacities. The investigation was conducted to evaluate efficacy in using ozonized oils (HOO) to neutralize the SARS-CoV-2 virus. Our interest in this approach was triggered by the serendipitous observation that no COVID-19 case was detected in cancer patients treated with HOO administered for prevention of cancer relapses. Indeed, cancer stem cells are addicted to antioxidants making them able to escape the therapeutic effects of chemo-radiotherapies.

Our therapeutic substance is an ozonized oil (HOO) which would be developed to release ozone intracellularly. There were several reasons to choose ozone. As a disinfectant, ozone is well known for killing viruses, especially RNA viruses [1,2]. In addition, a multiomics-based characterization of COVID-19’s vulnerability showed that ozone was a potentially effective drug [3]. Other reports also proposed its use for COVID-19 therapy [4,5,6,7,8]. The proposed therapeutic mechanisms included (a) inhibition of NFkB- and IL-1/6-driven inflammation [4]; (b) improvement of gas exchange and tissue respiration; and prevention of hypoxemia and multiorgan failure [7]. The recommended administration route was by auto-hemo-transfusion after blood ozonization. However, due to volatility of ozone, the timespan for antiviral efficacy would be limited. Furthermore, this invasive approach may not be suitable for use in healthy subjects nor in some infected patients. 

HOO is an oil-based ozone vector, which has been used for a long time in topical applications [9]. When administered in vivo by the oral route, it would complex with lipoprotein in the liver and then be distributed via the blood circulation with lungs as the first target organ. On the other hand, infection by COVID-19 occurs through the upper respiratory epithelium and nasopharyngeal mucosae. Therefore, to target these virus-entry tissues directly, we developed a novel hydrophilic preparation of HOO which would allow its delivery by aerosol and nasal sprays. This latter preparation is referred to as water soluble high ozonide oil (HOOws). Therefore, our systemic administration of HOO/HOOws for intracellular release of ozone represents a novel chemo-prophylactic tool to prevent COVID-19 infection in healthy subjects and a therapeutic tool to fight COVID-19 infection in affected patients. This tool is unspecific, thus being potentially active on all virus variants independently from SARS-CoV-2 antigen specificity. 

On the virus side, SARS-CoV-2 is highly sensitive to oxidation [10]. The sensitivity is related to the vulnerability lipid envelope of the virus, which is devoid of antioxidant defenses. Therefore, a key mechanism to efficacy is to take advantage of this viral deficiency and to kill the virus intracellularly, like what we have investigated using HOO and HOOws.

The efficacy of this approach was initially tested in vitro in cells sensitive to SARS-CoV-2 infection using the standard qPCR test to evaluate viral penetration inside the cells. Although existing COVID-19 assays are valuable, they have limitations. For example, rapid antigenic tests are fast but not entirely specific nor sensitive. PCR analyses for portions of viral RNA may identify degraded products rather than active infections. Furthermore, not all patients with a COVID-19 diagnosis have a positive qPCR-COVID-19 after two months for the same diagnosis. Consequently, infectious capacity of asymptomatic subjects can be underestimated by the existing tests, which contributes to epidemic spreading. Therefore, to enhance the determination of efficacy, a biological challenge test was developed by us to detect virus infectivity (or lack thereof) rather than presence or absence of the viral RNA. This assay can also be used to identify the biological capacity of COVID-19 to infect other subjects and to spread an active disease, such as poorly symptomatic but infective subjects who would play a key role in maintaining the epidemic.

In this study, we used the biological challenge test to test the efficacy of HOO and HOOws to neutralize SARS-CoV-2 and to prevent its penetration inside sensitive cells. Thereafter, the safety and efficacy of this approach was evaluated in human subjects and COVID-19 patients. 

## 2. Methods

### 2.1. In Vitro Studies

#### 2.1.1. Cell Culture for the Viral Challenge Experiments

The VERO C1008 (E6) African green monkey kidney cells (Vero) were certified by IZSLER (code BSCL87, Experimental Zoo-prophylactic Institute of Lombardia and Emilia Romagna Region, Ministry of Health, Brescia, Italy). These cells expressed much higher levels of angiotensin-converting enzyme 2 (ACE) on their outer membrane than most other cell types, e.g., human bronchial cells [11]. Routinely, these cells were maintained in semiconfluence cultures in our standard laboratory. The culture medium was a DMEM/fetal calf serum/Hepes buffer, and the cultures were kept inside 37 °C incubators with 5% CO_2_. 

For the challenge experiments, the culture medium for the Vero cells was changed into modified DMEM/fetal calf serum/Hepes buffer which allowed the cells to grow without CO_2_ [12], and the culture flasks were transferred from our routine laboratory to the BLS3 lab of the Research Center of the San Martino Hospital. For the challenge, SARS-CoV-2 containing oropharyngeal swab samples were taken from the freezer, thawed under a biosafety hood in a negative pressure room, and used for the challenge experiment in the BLS3 lab. 

#### 2.1.2. SARS-CoV-2 Challenge Experiments

An aliquot (0.5 mL) of the SARS-CoV-2 containing swap sample was dropped into the culture medium (DMEM/Hepes/FCS) of the Vero culture, each flask was gently mixed for 1 min and then incubated for 12 h at 37 °C. The 12 h time span was selected because it has been reported as the time when the highest level of virus penetrance into the cells would occur [13]. Oropharyngeal samples which were devoid of SARS-CoV-2 (qPCR negativity >40 amplification cycles) were used as negative controls.

After 12 h, the flasks were transferred from the incubator into a heated-hybridization oven (Bibby Stuart, Staffordshire ST15, OSA, UK) at 60 °C for 30 min to inactivate virus infectivity without altering viral RNA integrity and to detach cells from the flask. After inactivation, each flask was transferred to the biosafety hood where the cell-containing medium (12 mL) was collected into sterile tubes and the tubes were centrifuged at 3000× *g* for 15 min. The supernatant was discarded. Each cell pellet was resuspended, washed in molecular-grade physiological solution, and centrifuged twice. For each sample, the amount of RNA in each cell pellet was quantified by Qubit fluorescent probe analysis (Qubit 3.0 Fluorimeter, Life technologies, Qubit 3.0 Fluorimeter, Thermo Fisher Scientific, Carlsbad, CA, USA) and a standard RNA amount equal to those of the Cv19+ reference sample was used for RNA extraction and qPCR analyses. Each pellet was resuspended in RNAase-free molecular-grade water (1 mL) and frozen at −20 °C until RNA extraction.

#### 2.1.3. RNA Extraction and qPCR Analyses

The presence of viral RNA inside the challenged Vero cells was tested by qPCR using the SARS-CoV-2 RT-qPCR Reagent Kit (Perkin Elmer, Wathman, MA, USA). Samples were prepared using the automated Janus G3 workstation (Perkin Elmer, Wathman, MA, USA). Thawed samples (300 µL), composed of resuspended Vero cells, were mixed with a solution containing poly(A)RNA buffer and proteinase K solution (14 µL). RNA extraction was performed using the automated Chemagic workstation (Perkin Elmer, Wathman, MA, USA) and the magnetic-beads-based CheMagic extraction kit.

Three qPCR Taqman probes were used for testing a house-keeping gene (Ribonuclease P/MRP Subunit P30 [RPP30]) as internal control and the SARS-CoV-2 viral genes Orf1ab (Vic labeled) and N (FAM labeled). Purified RNA underwent PCR amplification according to the following cycles: 50 °C × 15 min, 95 °C × 2 min, 45 cycles at 95 °C × 3 s, and 60 °C × 30 s. PCR reactions were performed by Light Cycler 480II robotic machine (Roche) in a final volume of 20 μL.

#### 2.1.4. Prevention of SARS-CoV-2 Infection in Cell Culture

Various antivirus methods were tested for their ability to hamper SARS-CoV-2 penetration into the Vero cells. For each test, an aliquot (0.5 mL) of the same SARS-CoV-2 -containing samples which were used for the challenge test was treated with one of the following conditions:

(a) UV-C 254 nm radiation generated by LED, power 0.3 mW/cm^2^ (measured by Referenz Radiometer, Epigap Optoelekronik, GmbH, Bergkirchen, Germany) for 15 min, corresponding to a dose of 270 mJ/cm^2^.
(b) Hydrogen peroxide: Analytical grade sterile hydrogen peroxide (Sigma, Milan City, Italy) was added at a final concentration of 1% vol/vol and incubated at room temperature for 15 min.
(c) HOO (O3zone, GS Pharma, La Valletta, Malta): 0.5 mL was added to cultures and incubated at room temperature for 15 min. This ozonized oil was selected because (a) it had the highest level of ozonide available, i.e., >900 ozonides, and (b) it was the only ozonized oil among those tested which was able to penetrate inside pulmonary A549 cells (see below).
(d) HOOws (O3zone spray, GS Pharma, La Valletta, Malta): It contained water, lecithin, polysorbate 20, and ozonized peanuts oil. In addition, 0.5 mL of HOOws was added to cultures and incubated at room temperature for 15 min.
(e) HOO and HOOws in combination (1/1 vol/vol) incubated at room temperature for 15 min.

After the various treatments (or no treatment), samples were transferred into flasks containing Vero cells, incubated at 37 °C for a 12 h and then processed as previously reported for the challenge test. 

For this experiment, the negative and positive samples were untreated Vero E6 cells and nontreated medium containing the COVID-19 oropharyngeal swab, respectively. All experiments were performed in three independent replicates.

#### 2.1.5. Evaluation of Anti-Inflammatory Capacity of Ozonized Oils

Pulmonary alveolar macrophages can be activated inside the lung of COVID-19 infected patients triggering inflammation. Macrophage activation could cause complications, e.g., development of thromboembolic pneumonitis consequent to the release of pro-thrombotic factors, especially Thromboxane A2, from these cells when activated [14]. Since ozonized oils have anti-inflammatory capacity [15], this capacity was tested using HOO in immortalized murine macrophages (RAW264.7, IRCCS San Martino Biobank, Genoa, Italy). These cells were cultured for 24 in DMEM 75% *v*/*v* and FCS 25% *v*/*v*, and then activated by incubation with 10 ug/m *E. coli* lipo-polysaccharidic antigen (Lps, Sigma, Milan City, Italy) according to a procedure [16]. Cells were then either exposed to HOO (2 h pretreatment, 10% *v*/*v*) or, as sham control, to sunflower seed oil (2 h pretreatment, 10% *v*/*v*). Macrophage activation was determined by analyzing changes in morphology using standard microscopy.

#### 2.1.6. Evaluation of Ozonized Oil Penetration Inside Cultured Cells

SARS-CoV-2 replications occur intracellular; therefore, it is important to determine that HOO would also reach the intracellular compartment. This issue was explored by tracing labeled HOO (with red Nile dye, Sigma, Milan City, Italy) into cell cytoplasm as visualized by fluorescence microscopy. In this experiment, A549 human alveolar basal epithelial cells (ATCC CCL-185) were maintained in Dulbecco’s modified Eagle’s medium/F12 containing 10% fetal bovine serum, penicillin (100 U/mL), and streptomycin (100 mg/mL) and treated with labeled sunflower seed oil (sham-control, 2 h,10% *v*/*v*) or labeled HOO (2 h, 10% *v*/*v*).

### 2.2. In Vivo Studies

#### 2.2.1. Evaluation of Increased Oxygen Availability in the Lungs after HOO Treatment

In two healthy subjects, HOO effects on respiratory capacity before (T0) and after (T1) 1 week of HOO oral administration (3 cps × 2 × day) were evaluated. The maximal oxygen uptake (VO_2_max), VO_2_ at anaerobic threshold (VO_2_@AT), and the percentage of VO_2_max at anaerobic threshold (%VO_2_@AT) were used as criteria to assess oxygen availability. These subjects were healthy males, age 56 and 57 years, sedentary lifestyle, no drug consumption, and nonsmokers. 

The subjects participated in a cardiopulmonary exercise test (CPET) to determine a) VO_2_max (L/min and mL/kg/min; absolute and relative value, respectively); (b) VO_2_@AT (L/min and mL/kg/min); and (c) %VO_2_@AT. As a warm-up for the CPET, they were asked to run on a treadmill for 5 min at 7 km/h speed at 1% grade. Then, a strenuous exercise was performed by running with an increasing speed from 8 km/h with increments of 1 km/h for each minute till exhaustion. In addition, they performed the CPET with calibrated ergo-spirometer (Sensormedics, Viasys, CA, USA) to obtain cardiorespiratory parameters during the bouts, from warm-up to the end of the exercise. Expired gas samples were collected and analyzed breath by breath. VO_2_max was considered to be reached when at least 3 of the 4 following criteria were fulfilled: (i) a steady state of VO_2_ despite increasing running velocity (change in VO_2_ ≤ 150 mL/min at VO_2_max); (ii) final respiratory-exchange ratio (RER) exceeded 1.1; (iii) visible exhaustion; or (iv) a heart rate (HR) at the end of exercise (HRmax) equal to the predicted maximum (210—(0.65 × age)) [17]. The CPET tests were performed before (T0) the HOO administration and 1 week after (T1) the administration.

#### 2.2.2. Evaluation of Safety and Efficacy of Ozonized Oils in Human Patients

##### Study Design and Participants

The in vivo study was performed on *77* individuals including cancer patients without the infection (observational study 1), healthy subjects devoid of but at risk for COVID-19 infection (observational study 2), and individuals with the infection (intervention study). 

Under the approved protocol, three groups of subjects with our specific requirements (see inclusion and exclusion criteria) were randomly recruited from our community. All subjects had provided their consent to participate, undergone standard tests for COVID-19 infection status, and answered standard survey questions on age, gender, health conditions, etc. From all subjects, nose–mouth pharyngeal swabs were collected using the E-Swab collection kit (Copan, Brescia, Italy), placed into a vial individually which contained the company-supplied buffer, stored in our designated −20 °C freezers, and used within 2 weeks. 

Observational study 1 included 52 cancer patients treated with HOO as integrative support to anticancer therapies; inclusion criteria were previous cancer diagnosis (see Table 1), both genders, good health, age between 18 and 70 years, lack of participation in other clinical trials; exclusion criteria were uncontrolled cancer growth with a prognosis <6 months survival, hospitalization, cachexia, severe clinical condition, bone marrow failure, and liver failure. 

Observational study 2 included 21 healthy subjects treated for COVID-19 chemo-preventive purposes. Inclusion criteria were both genders, good health, age between 18 and 70 years, and lack of participation in other clinical trials. Exclusion criteria were pregnancy and breastfeeding, BMI ≥ 35 kg/m^2^, liver failure, chronic hepatitis, cirrhosis, and cholestatic liver diseases, any severe medical condition, alcohol and drug abuse, or history of known allergy to peanuts.

Intervention study included four COVID-19-affected patients treated for therapeutic purposes. Inclusion criteria (in addition to those previously reported) were SARS-Cov-2 qPCR positivity, COVID-19 severity score ≤5; exclusion criteria (in addition to those previously reported) were severe and critical COVID-19 pneumonia (COVID-19 severity score >5), patient connected to the ventilator, and blood oxygen saturation (SaO_2)_ less than 80%.

Consumptions of HOO and HOOws for these studies have been approved by the Health Ministry of Malta (approval number 0075/2020 according to EC1924/2006 issued on 17 March 2020). Within a 3-month duration, HOO was administered orally (1–3 cps × 3 × day), HOOws was administered by intranasal spray, two puffs (i.e., 100 μL) per nostril every 4 h.

##### Statistical Analyses

Differences between continuous numeric variables were tested by the nonparametric Mann–Whitney-U and Kruskal–Wallis tests. Differences between frequencies were tested by Chi-square analyses. Statistical significance of differences in disease incidence were evaluated by Mantel–Cox analysis. All statistical analyses were performed using the Stat view software (Statview, SAS Institute, Abacus Concept Inc., Berkeley, CA, USA).

## 3. Results

### 3.1. In Vitro Studies

#### 3.1.1. SARS-Cov-2 Challenge Test and qPCR Analyses 

qPCR detection of the house-keeping gene, which was used as internal control and as expression standard, was consistently present in all Vero cultures (untreated, treated with virus-negative swab samples, and treated with virus-positive swab samples cultures). For the Orf1 and N viral genes, the virus-negative controls did not reveal any positive amplification, whereas the virus-positive cultures showed amplifications for both genes at the 22th and 24th cycles (average 23th cycle), respectively. The data indicate successful infection of the Vero cells by SARS-Cov-2.

#### 3.1.2. Efficacy in Preventing SARS-Cov-2 Infection

**Figure 1:**
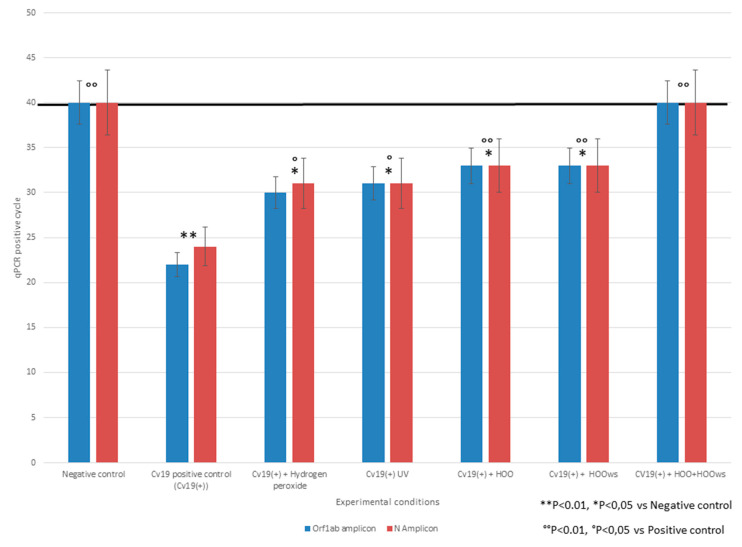
Number of qPCR positive amplification cycles for two SARS-CoV-2 viral genes (N, Orf1) under different prevention treatment protocols. Horizontal black line indicates the positivity threshold; samples negative at the 40th qPCR cycles were negative. Columns height is inversely related to the amount of SARS-CoV-2 penetrated inside Vero cells. All preventive treatments tested significantly decreased intracellular viral load. The only treatment able to restore, despite SARS-CoV2 presence in cell culture; the negative results obtained with negative control were HOO+HOOws.

The efficacy of hydrogen peroxide, UV, HOO, and HOOws in preventing SARS-CoV-2 penetration inside Vero cells was determined calculating the qPCR positivity threshold by quantifying intracellular load of viral-RNA. Efficacy was indicated by the delay in amplification positivity thresholds at qPCR for both the Orf1 and N genes as compared to the virus-positive cultures. The differences in qPCR threshold-detection cycles between the virus positive cultures and those treated with antiviral treatments were: hydrogen peroxide (four cycles), UV (five cycles), HOO (eight cycles), HOOws (nine cycles), and HOO+HOOws (23 cycles). These results are reported in Figure 1. According to these data, the rankings of efficacies in prevention of infection, in increasing order, were hydrogen peroxide, UV, HOO, HOOws, HOO pre-treatment, and HOO+HOOws. Indeed, HOO+HOOws was the only protocol which was capable of completely neutralizing COVID-19. The statistical significance of differences between qPCR cycles was calculated both versus negative and positive controls. The lack of difference between treated samples and negative control indicate a complete efficacy of the preventive treatment. This situation was achieved only by HOO/HOOws. Conversely, all preventive treatments significantly decreased intracellular viral load as compared to the positive control. Because no remarkable difference between results obtained for N and Orf1 probes was observed, statistical analyses considered the mean of these results for each experimental condition. 

#### 3.1.3. Evaluation of Anti-Inflammatory Capacity of Ozonized Oils

**Figure 2:**
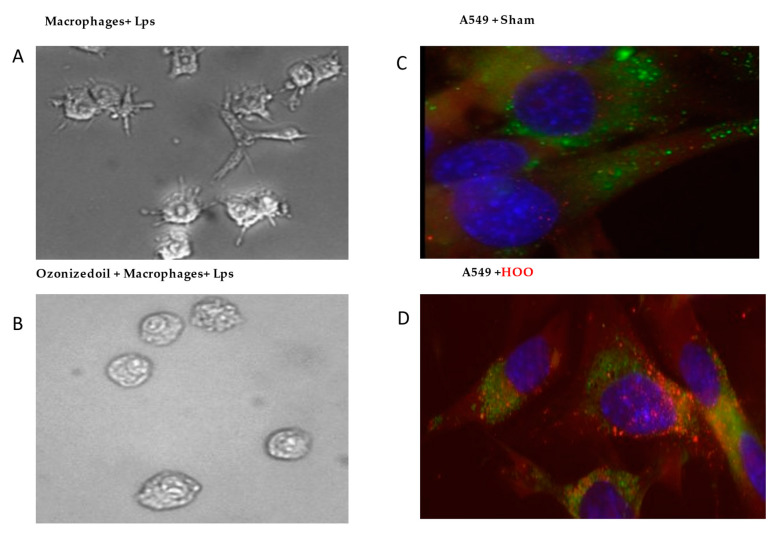
Left panel. Inhibition of pulmonary alveolar macrophage-activation by HOO. Panel (**A**), macrophages are activated in presence of Lps bacterial endotoxins changing their shape and emitting long pseudopods. Panel (**B**), macrophage activation does not occur despite the presence of Lps when cells are pretreated with ozonized oil (HOO). Right panel. Intracellular delivery of HOO (red labeled) in pulmonary cells. Panel (**C**), no penetration of red-labeled peanuts oil occurs in lung cells blue-stained for their nucleus and green-stained for their cytoplasmic membranes. Panel (**D**), Abundant penetration in cytoplasm of red-labeled ozonized oil (HOO) in lung cells.

Inflamed activated macrophages in culture usually demonstrate prismatic shape with pseudopods (Figure 2A). However, these events did not occur after macrophages were treated with HOO, despite the presence of high amounts (10 ug/mL) of the Lps activator. Indeed, these treated macrophages maintained their usual rounded shape without the emission of pseudopods (Figure 2B).

#### 3.1.4. Evaluation of Ozonized Oil’s Capacity to Penetrate inside Cultured Cells

After exposing pulmonary cells to un-ozonized (labeled) peanuts oil, no penetration was observed, as indicated by the lack of red dye intracellularly (Figure 2C). Conversely, a high abundance of red droplets was visible when cells were treated with labeled HOO. The results indicate that HOO was highly efficient in reaching the intracellular compartment (Figure 2D).

### 3.2. In Vivo Studies

#### Evaluation of Increased Oxygen Availability in the Lungs after Ozonized Oil Intake

In the examined subjects, VO_2_max values indicate an increase (T0: 38 ± 1.98; T1: 39.9 ± 1.41 mL/kg/min) after 1 week of HOO intake per os. Similarly, VO_2_ values at anaerobic threshold (VO_2_@AT) indicate an increase of 3.4% (T0: 31.55 ± 1.34; T1: 34.50 ± 1.56 mL/kg/min). Finally, percentage of VO_2_max at anaerobic threshold (%VO_2_@AT) increased by 4%. The data (means ± SD) are reported in Table 1. 

### 3.3. In Vivo Safety in Human Patients

During the past 6 months, 52 cancer patients received HOO oral treatment as integrative support to their anticancer therapies. During the treatment, liver functions (transaminase, bilirubin, etc.) and other standard haematochemical analyses were monitored, and no abnormal values were observed. No other obvious side effects were either observed or reported. The only minor adverse response was the rarely reported meteorism during the first 2 days of treatment in four patients. The inflammatory profile was measured analyzing C-reactive protein and speed of blood red-cell sedimentation after 1 h. For the C-reactive protein, values were at T0 (before HOO treatment) 0.9 ± 0.5 and at T1 (after HOO treatment) 0.3 ± 0.2 mg/100 mL (max normal value 0.5 mg/100 mL) (T1 vs. T0 *p* < 0.01). For the speed of blood red-cell sedimentation after 1 h, values were at T0 21.8 ± 3.1 and at T1 12.0 ± 1.4 mm (max normal value 16 mm) (T1 vs. T0 *p* < 0.01). These results are in line with the established anti-inflammatory capacity of ozone derivatives [15]. 

#### Anti-Viral Efficacy in Human Patients 

In the same 52 cancer patients, the efficacy of HOO in preventing COVID-19 infection was evaluated retrospectively. No infection was detected in the 6-month duration of the follow-up (Table 2). On the other hand, an infection incidence of 20% was expected, corresponding to at least 10 cases among the cancer patients. The expected frequency was estimated based on the actual incidence of COVID-19 infection in Italy and the high sensitivity of cancer patients to the infection. This difference (0% vs. 20% in 52 subjects) was statistically significant (*p* < 0.01). 

The efficacy of HOO in preventing COVID-19 infection was evaluated in 21 normal subjects who consumed HOO for 2 months as an integrative food supplement, and they carried on their normal daily activities. Due to the incidence of COVID-19 infection in Italy during the monitored period (i.e., 15% prevalence per 100 diagnostic tests performed), at least three of them would be expected to be infected within our study timeframe. However, none of the test subjects experienced COVID-19 infection, as demonstrated by the lack of any symptoms (fever, olfactory and test failure, cough, etc.), and by the negative antigen and molecular tests for COVID-19 infection. This difference (0% vs. 15% in 21 subjects) was statistically significant (*p* < 0.05).

Among the same 21 normal subjects, a strong evidence for the antiviral efficacy was shown in a frail 93-year-old female. She resided in a nursing home where COVID-19 outbreak did occur. Among the residents in the nursing home, one person in the same room died, and three others experienced severe pneumonitis and complications. Despite the extensive exposure to COVID-19 infection of this fragile subject, no symptoms occurred to her, and the weekly PCR molecular tests were also negative. 

Finally, therapeutic efficacy of HOO against COVID-19 was evaluated in four patients who were either diagnosed with having the infection by both clinical symptoms and molecular tests. 

The first patient was a 22-year-old female with infection on 15 August 2020. Her clinical symptoms included fever (39 °C), severe cough, thorax pain on cough, and loss of olfactory and taste function. Pharyngeal swab confirmed the diagnosis by detecting a high SARS-CoV-2 load with early cycle (<25th) qPCR positivity. She was administered four pills of HOO twice per day per os. After 5 days of treatment, all reported symptoms disappeared. In addition, thorax Rx confirmed the lack of any lung complications. The early recovery of olfactory and taste functions was unexpected, given the fact that these symptoms often persist for months after recovery. The second pharyngeal swab performed at 14 days since HOO treatment beginning was negative based on qPCR results.

The second patient was a frail 55-year-old male who had existing complications with COPD-related respiratory failure, obesity, and severe cardiovascular disease. He contracted COVID-19 infection together with pneumonitis, cough, fever (38.7 °C), and decreased O_2_ blood saturation down to 84%. After 4 days of HOO treatment, the patient’s fatigue and fever disappeared together with recovery of olfactory and taste capacities. Importantly, O_2_ blood saturation was restored to 98%. qPCR tests for SARS-CoV-2 performed 7 and 14 days after beginning HOO treatment were negative.

The third patient was a 54-year-old female who was the wife of the second patient being therefore heavily exposed to the SARS-CoV-2 virus. HOO treatment was started 2 days after her husband was diagnosed with the infection. Although she had no clinical symptoms for the infection at that time, thorax Rx revealed the presence of asymptomatic lung pneumonitis amenable to COVID-19 features. After the HOO treatment, the molecular test for COVID-19 diagnosis was negative, and no symptoms for COVID-19 infection appeared. Therefore, her infection with COVID-19 was possibly prevented.

The fourth patient was a 52-year-old female working in the nursing home where our abovementioned 93-year-old female lived and where the COVID-19 outbreak occurred. She had moderate COVID-19 infection symptoms, which were confirmed by positive qPCR molecular test. HOO was started immediately, and after 5 days, all symptoms disappeared. The qPCR molecular test performed 10 days after beginning HOO treatment provided negative result.

In summary, a total of 77 subjects received HOO administration either for chemoprophylaxis in uninfected subjects (*n* = 73, 52 cancer patients and 21 healthy subjects) or for therapeutic purposes in COVID-19 infected patients (*n* = 4). The results indicate that no COVID-19 infection was detected in the uninfected subjects, and complete recovery with negative qPCR tests was observed in the four infected patients. These results are summarized in Table 2. 

## 4. Discussion

With the escalating COVID-19 pandemic, a variety of effective prevention and intervention protocols is being developed and is urgently needed to combat the disease. Our investigations, using cell culture, normal subjects, cancer patients, and COVID-19-infected patients, indicate that ozonized oil can be used as novel chemoprophylaxis and therapy against COVID-19 infection. Large controlled studies and clinical trials are required to substantiate these findings.

For our investigation, the virus challenge test was developed and was shown to be sensitive and specific in detecting the ability of SARS-CoV2 to infect sensitive (i.e., ACE2 expressing) cells. For prevention, HOO when used in combination with HOOws was more effective than some common antiviral treatments. The combined treatment was so effective that it fully neutralized SARS-CoV2 infectivity because no virus was detected inside the cells despite their exposure to a very high dose of viral load. This finding was obtained in pulmonary cells, i.e., SARS-Cov-2 target cells. However, it could be also proven in other different type of cells to corroborate this result.

HOO is an oil-based ozone vector. When administered in vivo by the oral route, it is complexed with lipoprotein in the liver and then distributed via the general circulation, with lungs as the first target organ. However, infection from COVID-19 is mainly via the upper respiratory epithelium. Indeed, the first entry tissue for SARS-Cov-2 infection is represented by nasal mucosa [18]. To directly target these entry tissues, a novel hydrophilic preparation of HOO was developed by us and is labeled as water soluble high ozonide oil (HOOws). Specifically, the preparation allows its delivery by aerosol and nasal spray. 

Despite our limited sample sizes, our data provide valuable and novel evidence to indicate that the combination of HOO and HOOws was highly effective in preventing COVID-19 infection. The prevention mechanisms are mediated by neutralizing the virus both in intracellular and extracellular environments, inhibiting intracellular viral replication and blocking extracellular spreading of virions, without obvious side-effects. 

**Figure 3:**
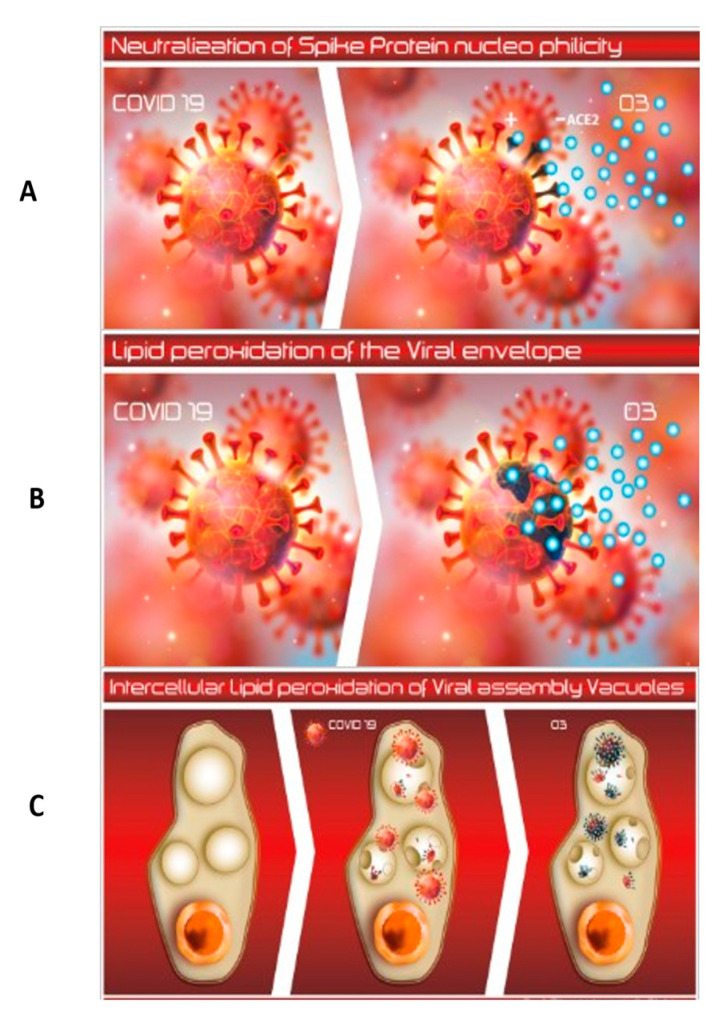
(**A**–**C**). Possible mechanisms for sensitivity of SARS-CoV-2 19 virus to HOO. Panel (**A**) Neutralization of spike proteins; HOO oxidation blocks the sites of the spike protein used by SARS-Cov-2 to bind cell receptor ACE2; this situation is highlighted by the darkening of spike protein when treated with HOO (light blue circles). Panel (**B**) Peroxidation of the lipid viral envelope; due to the low spike-protein density, and wide sections of the SARS-Cov-2 lipid envelope are exposed to the interaction with HOO; this situation results in the peroxidation of the viral lipid envelope, as envisaged by the darkening of this structure when interacting with HOO (light blue circles). Panel (**C**) HOO has a unique ability to penetrate inside cell cytoplasm where the viral replication cycle occurs hidden from extracellular disinfectants; HOO is able to neutralize intracellular viral assembly oxidizing viral components inside the intracellular environment (darkening of intracellular viral fragments when interacting with HOO light-blue circles). From left to right: normal cell, cell infected by SARS-Cov-2, and cell infected by SARS-Cov-2 treated with HOO.

The observed effectiveness of HOO is most likely based on the unique structure, morphology, and composition of the SARS-CoV2 virus. SARS-Cov-2 sensitivity to UV disinfection is controversial because RNA viruses do not contain thymine (T), the main molecular target of UV-C, but uracil (U). Indeed, UV-C genotoxicity is exerted by forming intrastrand T-T cyclobutane dimers [19]. However, SARS-Cov-2 has been reported to be very sensitive to 222 nm and, to a lesser extent, 254 nm UVC radiations [20,21]. It should be noted that, besides being genotoxic, UV light is also an oxidizing agent. Human coronaviridae were reported to be sensitive to hydrogen peroxide disinfection in the same levels of Glutardialdehyde [10]. One reason for the sensitivity of SARS-CoV-2 to oxidizing agents is that the virus lacks any antioxidant defenses. Another is the presence of chemical structures very sensitive to oxidation at the terminal part of the spike-protein which binds the ACE2 cell receptor. The terminal region of the spike-protein is enriched with thiol-rich amino-acids, i.e., cysteine, whose sulfhydryl (–SH) sites are highly sensitive to oxidation [7]. These structures of the viral-spike proteins can be neutralized by oxidizing agents, such as HOO. This neutralization would hamper its virus binding with the ACE2 receptor thus blocking intracellular virus penetration (Figure 3A).

Under our experimental conditions, hydrogen peroxide was not particularly effective in neutralizing SARS-CoV-2. This situation is amenable to the peculiar characteristic of this virus and to its high lipophilicity, making it a difficult target for hydrophilic disinfectant such as hydrogen peroxide. Indeed, the ratio between the protein content (spike proteins) and the lipid content (envelope) is dramatically low in SARS-CoV-2 19 as compared to other RNA viruses. As an example, in the external surface of flu *Orthomyxovirus*, there are 1 protein per 100 nm^2^ of membrane lipid surface. Due to the high density of outside proteins (hemagglutinin and neuraminidase), this virus is referred to as “chestnut hedgehog” virus. By comparison, this ratio dramatically drops by 10-fold in SARS-CoV-2 19 virus to 1 protein per 1000 nm^2^ only [22]. Furthermore, SARS-CoV-2 19 spike proteins are highly flexible and can fold and move along exposing to the outside large sections of the underlying lipid envelope [23]. Such a structure explains the high lipophilicity of SARS-CoV-2 which exposes large sections of the envelope for the interaction with target cells. Accordingly, SARS-CoV-2 interacts earlier and more readily with nerves, neurons, and the central nervous system, which are typically highly lipophilic, than other airborne RNA viruses such as *Orthomyxoviridae* [24,25]. The early neurotropism of SARS-CoV-2 19 causes the early symptoms which are related to its penetration into olfactory nerve (to cause loss of olfactory and taste capabilities) and across haemato-encephalic barrier [26].

Since SARS-CoV-2 exposes a wide portion of its lipid envelope to the outside, without a screen provided by a dense layer of spike proteins, it becomes highly sensitive to ethanol-containing disinfectants. Indeed, ethanol, which usually fixes but does not kill viruses, due to its lipid-solvent actions, is the most effective disinfectant against SARS-CoV-2 [10].

The unique structure of SARS-CoV-2, as described earlier, indicates its high sensitivity to oxidizing disinfectants that are also lipophilic. This situation is clearly demonstrated by our use of HOO and HOOws. Their effectiveness is likely based on the following scenario: Due to their lipid component (saturated fatty acids), these compounds easily target the unscreened lipophilic envelope of the virus. When the target is reached, both HOO and HOOws would release ozone and reactive oxygen species to induce lipid peroxidation in the sensitive virus that is devoid of any antioxidant defenses. These activities would destroy the envelope and neutralize the virus, thus eliminating infectivity and consequences from the infection. This mechanism of action is reported in Figure 3B. 

Another possible explanation for the anti-COVID efficacy of HOO is the ability of this compound to interfere with the formation and dynamics of lipid vacuoles which protect the virus during the intracellular production and assembly of the whole virions [27]. As demonstrated in our above-reported in vitro studies, HOO has a unique ability to penetrate inside cell cytoplasm where the viral replication cycle occurs hidden from extracellular disinfectants. Thus, HOO is able to neutralize intracellular viral assembly oxidizing viral components inside the intracellular environment. This mechanism of actions is summarized in Figure 3C.

This mechanism also explains the synergistic effects between HOO and HOOws: with HOO being lipophilic and targeting SARS-CoV-2 in both extra- and intra-cellular compartments; with HOOws being hydrophilic and targeting SARS-CoV-2 19 virus in the extracellular compartment. Indeed, the combined administration of HOO and HOOws was found to be highly effective in preventing and attenuating COVID-19 infection in the 77 normal individuals and patients without adverse or side effects. It is important to point out that our group of subjects is composed of many high-risk individuals: four COVID-infected patients, 52 cancer patients who are susceptible to infection [28], and normal senior citizens in an infectious environment. 

A highly relevant observation is the disappearance of symptoms in just 5 days after HOO treatment and of negative qPCR results after 7–10 days among the four COVID-infected patients. The quick recovery was encouraging but unexpected because COVID-19 patients usually display positive qPCR results up to 60 days after recovery [29]. On the other hand, the early disappearance of clinical symptoms was likely due to HOO’s anti-inflammatory and macrophage-inhibiting effects, which also explains the lack of thrombo-embolic complications among our patients. In addition, HOO’s capacity to increase oxygen availability in the lungs would counter pulmonary damage from COVID-19, as demonstrated in the fragile patient. Such an increased oxygen supply would also be of great benefit to COVID-19 patients who are also affected by severe pneumonia. Indeed, COVD-19 infection cause an interstitial pneumonia [30] hampering oxygen absorption by the alveolar endothelium despite the delivery of oxygen through the respiratory system, as performed for therapeutic purposes. Accordingly, the possibility of directly increasing oxygen tissue availability as performed by HOO is of relevance to improve the prognosis of COVID-19 patients. The limit of our study is that analysis of oxygen tissue availability and aerobic threshold has been performed in healthy subjects and not in COVID-19 patients. Unfortunately, COVID-19 patients cannot undergo the physical endurance activity required to perform the aerobic threshold analysis. However, the findings obtained in COVID-19 patients indicate that blood oxygen saturation is remarkably increased after HOO treatment. To our knowledge, this is the first study to develop an innovative carrier for ozone which would release ozone intracellularly and to evaluate its clinically relevant antiviral activities and efficacy.

Our study has limitations. The findings are not validated for a double-blind clinical study but an observational study. In this study efforts have been focused on demonstrating the mechanisms explaining the antiviral efficacy of ozonized oils against COVID-19. Further clinical studies from other clinical centers are required to substantiate the herein presented clinical results. The setting up of a double-blind randomized clinical trial as performed in hospital ward assisting COVID-19 patients is required to prove or deny the clinical efficacy of the proposed approach in an adequate number of patients.

## 5. Conclusions

Our novel and effective HOO/HOOws treatment protocol against COVID-19 is highly encouraging. Due to their naturally nontoxic status, HOO/HOOws can be used as chemo-prophylactic treatment against COVID-19 infection in different infectious environments: occupational (medical doctors, nurses) or familiar (relatives or cohabitants) conditions. In addition, for infected patients, HOO/HOOws can be used as complimentary therapeutic treatment for COVID-19 infection, without the need for any modifications of the established standard therapeutic protocols. This complimentary treatment is potentially helpful to (a) decrease the severity of the diseases, thus lowering the number of patients requiring high-intensity therapies and for (b) faster recovery and time spent in hospitals. Therefore, clinicians may adopt the use of our protocol for prevention and intervention of COVID infection. Randomized controlled clinical trials will be set up to definitively determine the effectiveness of this treatment in preventing SARS-Cov-2 infection and COVID-19 complications. With the collection of additional clinical results, efficacy of HOO/HOOws treatment will be better understood, and enhanced protocol will be used against the pandemic.

## Figures and Tables

**Table 1:** Evaluation of increased oxygen availability in human lung after ozonized oil intake.

T0 PRE-TREATMENT TEST	VO2 Max (mL/kg/min)	VO2 (L/min)	VO2 Threshold (mL/kg/min)	VO2 Threshold (L/min)	% VO2 Max in Threshold
Subject 1	39.4	3.19	32.5	2.62	82
Subject 2	36.6	2.42	30.6	2.07	86
**T1 POST-TREATMENT TEST**					
Subject 1	40.9	3.27	35.6	2.94	90
Subject 2	38.9	2.63	33.4	2.08	87
**T1-T0 delta after HOO TREATMENT**					
Subject 1	+1.5	+0.08	+2.9	+0.32	+8
Subject 2	+2.3	+0.21	+2.8	+0.01	+1

**Table 2:** Subjects undergoing ozonized oil (HOO) treatment for either chemo-prophylactic (*n* = 52 cancer patients +21 normal individuals) or therapeutic purposes (*n* = 4 infected patients) of COVID-19 infection.

Gender	Age	Previous Diseases	COVID-19 Infection	Clinical Outcome
Therapeutic purpose				
Female	22	None	Yes	Recovery
Male	55	COPD, Vascular ischemia	Yes	Recovery
Female	54	None	Yes	Recovery
Female	52	None	Yes	Recovery
Chemo-prophylactic purpose. Cancer patients				
Male	47	Brain cancer (glioblastoma)	No	No Covid-19 infection
Male	40	Brain cancer (glioblastoma)	No	No Covid-19 infection
Male	19	Brain cancer (glioblastoma)	No	No Covid-19 infection
Female	11	Brain cancer (glioblastoma)	No	No Covid-19 infection
Male	48	Brain cancer (glioblastoma)	No	No Covid-19 infection
Male	33	Brain cancer (glioblastoma)	No	No Covid-19 infection
Male	40	Brain cancer (glioblastoma)	No	No Covid-19 infection
Female	37	Brain cancer (glioblastoma)	No	No Covid-19 infection
Female	40	Brain cancer (glioblastoma)	No	No Covid-19 infection
Female	50	Breast cancer	No	No Covid-19 infection
Female	56	Breast cancer	No	No Covid-19 infection
Female	60	Breast cancer	No	No Covid-19 infection
Female	69	Breast cancer	No	No Covid-19 infection
Female	65	Breast cancer	No	No Covid-19 infection
Female	55	Breast cancer	No	No Covid-19 infection
Female	52	Breast cancer	No	No Covid-19 infection
Female	70	Breast cancer	No	No Covid-19 infection
Male	57	Colon cancer	No	No Covid-19 infection
Female	61	Colon cancer	No	No Covid-19 infection
Male	53	Colon cancer	No	No Covid-19 infection
Female	57	Colon cancer	No	No Covid-19 infection
Male	78	Kidney cancer	No	No Covid-19 infection
Male	73	Bladder cancer	No	No Covid-19 infection
Female	82	Non-Hodgkin Lymphoma	No	No Covid-19 infection
Male	54	Non-Hodgkin Lymphoma	No	No Covid-19 infection
Male	81	Lung cancer (NSCLC)	No	No Covid-19 infection
Male	58	Lung cancer (NSCLC)	No	No Covid-19 infection
Female	55	Lung cancer (SCLC)	No	No Covid-19 infection
Male	27	Lung cancer (SCLC)	No	No Covid-19 infection
Male	79	Lung cancer (NSCLC)	No	No Covid-19 infection
Male	76	Lung cancer (NSCLC)	No	No Covid-19 infection
Female	74	Ovarian cancer	No	No Covid-19 infection
Female	75	Ovarian cancer	No	No Covid-19 infection
Female	66	Ovarian cancer	No	No Covid-19 infection
Female	28	Womb cancer	No	No Covid-19 infection
Female	62	Pancreas cancer	No	No Covid-19 infection
Female	78	Pancreas cancer	No	No Covid-19 infection
Male	72	Pancreas cancer	No	No Covid-19 infection
Female	58	Pancreas cancer	No	No Covid-19 infection
Male	63	Pancreas cancer	No	No Covid-19 infection
Female	79	Pancreas cancer	No	No Covid-19 infection
Male	60	Pancreas cancer	No	No Covid-19 infection
Male	67	Pancreas cancer	No	No Covid-19 infection
Male	71	Prostate cancer	No	No Covid-19 infection
Male	80	Prostate cancer	No	No Covid-19 infection
Male	83	Prostate cancer	No	No Covid-19 infection
Male	58	Prostate cancer	No	No Covid-19 infection
Male	61	Prostate cancer	No	No Covid-19 infection
Female	92	Skin cancer (basal cell carcinoma)	No	No Covid-19 infection
Male	70	Oral cancer (squamous cell carcinoma)	No	No Covid-19 infection
Male	89	Skin cancer (basal cell carcinoma)	No	No Covid-19 infection
Male	77	Skin cancer (angiosarcoma)	No	No Covid-19 infection
Chemo-prophylactic purpose. Healthy subjects				
Female	32	None	No	No Covid-19 infection
Female	12	None	No	No Covid-19 infection
Female	18	None	No	No Covid-19 infection
Male	72	None	No	No Covid-19 infection
Female	45	None	No	No Covid-19 infection
Female	32	None	No	No Covid-19 infection
Female	38	None	No	No Covid-19 infection
Male	45	None	No	No Covid-19 infection
Male	59	None	No	No Covid-19 infection
Male	64	None	No	No Covid-19 infection
Female	49	None	No	No Covid-19 infection
Female	93	None	No	No Covid-19 infection
Male	61	None	No	No Covid-19 infection
Male	52	None	No	No Covid-19 infection
Male	34	None	No	No Covid-19 infection
Female	36	None	No	No Covid-19 infection
Female	48	None	No	No Covid-19 infection
Male	62	None	No	No Covid-19 infection
Male	46	None	No	No Covid-19 infection
Female	51	None	No	No Covid-19 infection
Male	80	None	No	No Covid-19 infection

## Data Availability

The datasets used and/or analysed during the current study are available from the corresponding author on reasonable request.

## Abbreviations

HOO: ozonized oils at high ozonides; HOOws: water-soluble ozonized oils at high ozonides.

## References

[1] Jiang H.J., Chen N., Shen Z.Q., Jing Y., Gang Q.Z., Jing M., Wei Y.Z., Yang S.D., Wang H.R., Wei W.X. (2019). Inactivation of Poliovirus by Ozone and the Impact of Ozone on the Viral Genome. Biomed. Environ. Sci..

[2] Torrey J., von Gunten U., Kohn T. (2019). Differences in Viral Disinfection Mechanisms as Revealed by Quantitative Transfection of Echovirus 11 Genomes. Appl Environ. Microbiol..

[3] Barh D., Tiwari S.E., Weener M.E., Azevedo V., Góes-Neto A., Gromiha M.M., Ghosh P. (2020). Multi-omics-based identification of SARS-CoV-2 infection biology and candidate drugs against COVID-19. Comput Biol Med..

[4] Martínez-Sánchez G., Schwartz A., Donna V.D. (2020). Potential Cytoprotective Activity of Ozone Therapy in SARS-CoV-2/COVID-19. Antioxidants.

[5] Zheng Z., Dong M., Hu K. (2020). A preliminary evaluation on the efficacy of ozone therapy in the treatment of COVID-19. J. Med. Virol..

[6] Valdenassi L., Franzini M., Ricevuti G., Rinaldi L., Galoforo A.C., Tirelli U. (2020). Potential mechanisms by which the oxygen-ozone (O2-O3) therapy could contributeto the treatment against the coronavirus COVID-19. Eur. Rev. Med. Pharmacol. Sci..

[7] Fernández-Cuadros M.E., Albaladejo-Florín M.J., Peña-Lora D., Álava-Rabasa S., Pérez-Moro O.S. (2020). Ozone (O3) and SARS-CoV-2: Physiological Bases and Their Therapeutic Possibilities According to COVID-19 Evolutionary Stage. SN Compr. Clin. Med..

[8] Franzini M., Valdenassi L., Ricevuti G., Chirumbolo S., Depfenhart M., Bertossi D., Tirelli U. (2020). Oxygen-ozone (O 2-O 3) immunoceutical therapy for patients with COVID-19. Preliminary evidence reported. Int. Immunopharmacol..

[9] Ugazio E., Tullio V., Binello A., Tagliapietra S., Dosio F. (2020). Ozonated Oils as Antimicrobial Systems in Topical Applications. Their Characterization, Current Applications, and Advances in Improved Delivery Techniques. Molecules.

[10] Kampf G., Todt D., Pfaender S., Steinmann E. (2020). Persistence of coronaviruses on inanimate surfaces and their inactivation with biocidal agents. J. Hosp. Infect..

[11] Kam Y.W., Okumura Y., Kido H., Ng L.F., Bruzzone R., Altmeyer R. (2009). Cleavage of the SARS coronavirus spike glycoprotein by airway proteases enhances virus entry into human bronchial epithelial cells in vitro. PLoS ONE.

[12] Calzia D., Ottaggio L., Cora A., Chiappori G., Cuccarolo P., Cappelli E., Izzotti A., Tavella S., Degan P. (2020). Characterization of C2C12 cells in simulated microgravity: Possible use for myoblast regeneration. J. Cell Physiol..

[13] Keyaerts E., Vijgen L., Maes P., Neyts J., Van Ranst M. (2005). Growth kinetics of SARS-coronavirus in Vero E6 cells. Biochem. Biophys. Res. Commun..

[14] Williams J.G., Maier R.V. (1992). Ketoconazole inhibits alveolar macrophage production of inflammatory mediators involved in acute lung injury (adult respiratory distress syndrome). Surgery.

[15] Tartari A.P.S., Moreira F.F., Pereira M.C.D., Carraro E., Cidral-Filho F.J., Salgado A.I., Kerppers I.I. (2020). Anti-inflammatory Effect of Ozone Therapy in an Experimental Model of Rheumatoid Arthritis. Inflammation.

[16] Cao Y., Chen J., Ren G., Zhang Y., Tan X., Yang L. (2019). Punicalagin Prevents Inflammation in LPS-Induced RAW264.7 Macrophages by Inhibiting FoxO3a/Autophagy Signaling Pathway. Nutrients.

[17] Thevenet D., Tardieu-Berger M., Zouhal H., Jacob C., Abderrahman B.A., Prioux J. (2007). Influence of exercise intensity on time spent at high percentage of maximal oxygen uptake during an intermittent session in young endurance-trained athletes. Eur. J. Appl. Physiol..

[18] Sungnak W., Huang N., Bécavin C., Berg M., Queen R., Litvinukova M., Talavera-López C., Maatz H., Reichart D., Sampaziotis F. (2020). HCA Lung Biological Network. SARS-CoV-2 entry factors are highly expressed in nasal epithelial cells together with innate immune genes. Nat. Med..

[19] Kurosaki Y., Abe H., Morioka H., Hirayama J., Ikebuchi K., Kamo N., Nikaido O., Azuma H., Ikeda H. (2003). Pyrimidine dimer formation and oxidative damage in M13 bacteriophage inactivation by ultraviolet C irradiation. Photochem. Photobiol..

[20] Hessling M., Haag R., Sieber N., Vatter P. (2021). The impact of far-UVC radiation (200-230 nm) on pathogens, cells, skin, and eyes—A collection and analysis of a hundred years of data. GMS Hyg. Infect. Control..

[21] Heilingloh C.S., Aufderhorst U.W., Schipper L., Dittmer U., Witzke O., Yang D., Zheng X., Sutter K., Trilling M., Alt M. (2020). Susceptibility of SARS-CoV-2 to UV irradiation. Am. J. Infect. Control..

[22] Ke Z., Oton J., Qu K., Cortese M., Zila V., McKeane L., Nakane T., Zivanov J., Neufeldt C.J., Cerikan B. (2020). Structures and distributions of SARS-CoV-2 spike proteins on intact virions. Nature.

[23] Turunova B., Sikora M., Schurman C., Hagen W.J., Welsch S., Blanc F.E.C., von Bülow S., Gecht M., Bagol K., Hörner C. (2020). In situ structural analysis of SARS-CoV-2 spike reveas flexibility mediated by three hinges. Science.

[24] Iadecola C., Anrather J., Kamel H. (2020). Effects of COVID-19 on the Nervous System. Cell.

[25] Mao L., Jin H., Wang M., Hu Y., Chen S., He Q., Chang J., Hong C., Zhou Y., Wang D. (2020). Neurologic Manifestations of Hospitalized Patients With Coronavirus Disease 2019 in Wuhan, China. JAMA Neurol..

[26] Buzhdygan T.P., DeOre B.J., Baldwin-Leclair A., Bullock T.A., McGary H.M., Khan J.A., Razmpour R., Hale J.F., Galie P.A., Potula R. (2020). The SARS-CoV-2 spike protein alters barrier function in 2D static and 3D microfluidic in-vitro models of the human blood-brain barrier. Neurobiol. Dis..

[27] Caldas L.A., Carneiro F.A., Hiha L.M., Monteiro F.L., da Silva G.P., da Costa L.J., Durigon E.L., Tanuri A., de Souza W. (2020). Ultrastructural analysis of SARS-CoV-2 interactions with the host cell via high resolution scanning electron microscopy. Sci. Rep..

[28] Oh W.K. (2020). COVID-19 infection in cancer patients: Early observations and unanswered questions. Ann. Oncol..

[29] Cento V., Colagrossi L., Nava A., Lamberti A., Senatore S., Travi G., Rossotti R., Vecchi M., Casati O., Matarazzo E. (2020). Persistent positivity and fluctuations of SARS-CoV2-RNA in clinically recovered COVID-19 patients. J. Infect..

[30] Machhi J., Herskovitz J., Senan A.M., Dutta D., Nath B., Oleynikov M.D., Blomberg W.R., Meigs D.D., Hasan M., Patel M. (2020). The Natural History, Pathobiology, and Clinical Manifestations of SARS-CoV-2 Infections. J. Neuroimmune Pharmacol..

